# Therapeutic inhibition of USP7-PTEN network in chronic lymphocytic leukemia: a strategy to overcome *TP53* mutated/deleted clones

**DOI:** 10.18632/oncotarget.16348

**Published:** 2017-03-17

**Authors:** Giovanna Carrà, Cristina Panuzzo, Davide Torti, Guido Parvis, Sabrina Crivellaro, Ubaldo Familiari, Marco Volante, Deborah Morena, Marcello Francesco Lingua, Mara Brancaccio, Angelo Guerrasio, Pier Paolo Pandolfi, Giuseppe Saglio, Riccardo Taulli, Alessandro Morotti

**Affiliations:** ^1^ Department of Clinical and Biological Sciences, University of Turin, San Luigi Gonzaga Hospital, Orbassano, Italy; ^2^ Division of Internal Medicine - Hematology, San Luigi Gonzaga Hospital, Orbassano, Italy; ^3^ Division of Hematology, Azienda Ospedaliera, Mauriziano, Torino, Italy; ^4^ Division of Pathology, San Luigi Hospital, Orbassano, Italy; ^5^ Department of Oncology, University of Turin, San Luigi Gonzaga Hospital, Orbassano, Italy; ^6^ Department of Molecular Biotechnology and Health Sciences, University of Torino, Torino, Italy; ^7^ Cancer Genetics Program, Beth Israel Deaconess Cancer Center, Department of Medicine and Pathology, Beth Israel Deaconess Medical Center, Harvard Medical School, Boston, MA, USA

**Keywords:** chronic lymphocytic leukemia, USP7, PTEN, miR181, miR338

## Abstract

Chronic Lymphocytic Leukemia (CLL) is a lymphoproliferative disorder with either indolent or aggressive clinical course. Current treatment regiments have significantly improved the overall outcomes even if higher risk subgroups - those harboring *TP53* mutations or deletions of the short arm of chromosome 17 (del17p) - remain highly challenging. In the present work, we identified USP7, a known de-ubiquitinase with multiple roles in cellular homeostasis, as a potential therapeutic target in CLL. We demonstrated that in primary CLL samples and in CLL cell lines USP7 is: *i)* over-expressed through a mechanism involving miR-338-3p and miR-181b deregulation; *ii)* functionally activated by Casein Kinase 2 (CK2), an upstream interactor known to be deregulated in CLL; *iii)* effectively targeted by the USP7 inhibitor P5091. Treatment of primary CLL samples and cell lines with P5091 induces cell growth arrest and apoptosis, through the restoration of PTEN nuclear pool, both in *TP53-*wild type and -null environment. Importantly, PTEN acts as the main tumor suppressive mediator along the USP7-PTEN axis in a p53 dispensable manner. In conclusion, we propose USP7 as a new druggable target in CLL.

## INTRODUCTION

Chronic lymphocytic leukemia (CLL) is a lymphoproliferative disorder with either an indolent clinical course and long survival time or aggressive behavior in a smaller proportion of cases [1–6]. Standard immuno-chemotherapy regimens achieve a 60-70% overall response rate, with only a fraction of treated patients obtaining a complete hematological remission, while the median time to treatment failure is around 18-20 months. Unsatisfactory therapeutic options still exist for higher risk groups, and in particular those harboring *TP53* mutations or deletion of the short arm of chromosome 17 (del17p) [7, 8]. In addition to chemotherapy and anti-CD20 immunotherapy, molecular insights into CLL pathogenesis and maintenance allowed to identify novel drugs to target a variety of signaling routes to enter the clinical arena [9]. These include PI3K inhibitors (e.g. *idelalisib*) [10], BTK inhibitors (*ibrutinib*, *acalabrutinib*) [11, 12] and BCL2 inhibitors (*venetoclax*, *navitoclax*) [13, 14]. Del17p has been reported to be present in ~7% of chemo-naïve CLL cases and 20-40% of relapsed patients and is routinely used as a prognostic marker, representing the sub-population facing an unmet clinical need. Furthermore, idelalisib (*Zydelig*) [15] and ibrutinib (*Imbruvica*) [16] have been FDA-licensed for CLL harboring del17p following optimistic results in randomized controlled trials. Some of these drugs are directed to PI3K pathway suggesting its essential role for the maintenance of CLL malignant phenotype irrespective of *TP53* status.

Recently, Chauhan *et al*. showed that pharmacological inhibition of the de-ubiquitinase USP7 strongly induces apoptosis in multiple myeloma cells resistant to conventional and bortezomib-based therapies [17]. USP7 promotes the de-ubiquitination of various targets with consequent changes in their protein levels and cellular compartmentalization and behaves as a central rheostat in the definition of cell survival. USP7 plays an essential role in tumorigenesis through the inactivation of three major tumor suppressors: p53 [18], PTEN [19, 20] and FoxO [21]. By affecting either their cellular compartmentalization or adjusting their protein levels, USP7 influences the cellular fate, balancing survival and cell death. Both p53 [22] and PTEN [23–28] are known to play important tumor suppressive roles in CLL pathogenesis. In particular, while *TP53* is in most cases genetically inactivated through point mutations or deletion and correlates with resistance to standard treatments and poor prognosis, PTEN has been shown to be functionally inactivated through tail-phosphorylation by the CLL relevant Protein Kinase 2 (CK2) [26, 27, 29, 30]. Here we investigate the mechanisms of USP7 regulation in CLL, explore the functional role of USP7 in the broader context of its signaling partners (i.e. the USP7-PTEN network) and provide evidences supporting its potential therapeutic exploitation. Finally, we discuss the ability of USP7 inhibitor to effectively target CLL cells regardless of their *TP53* status.

## RESULTS

### USP7 is strongly up-regulated in CLL samples

**Figure 1:**
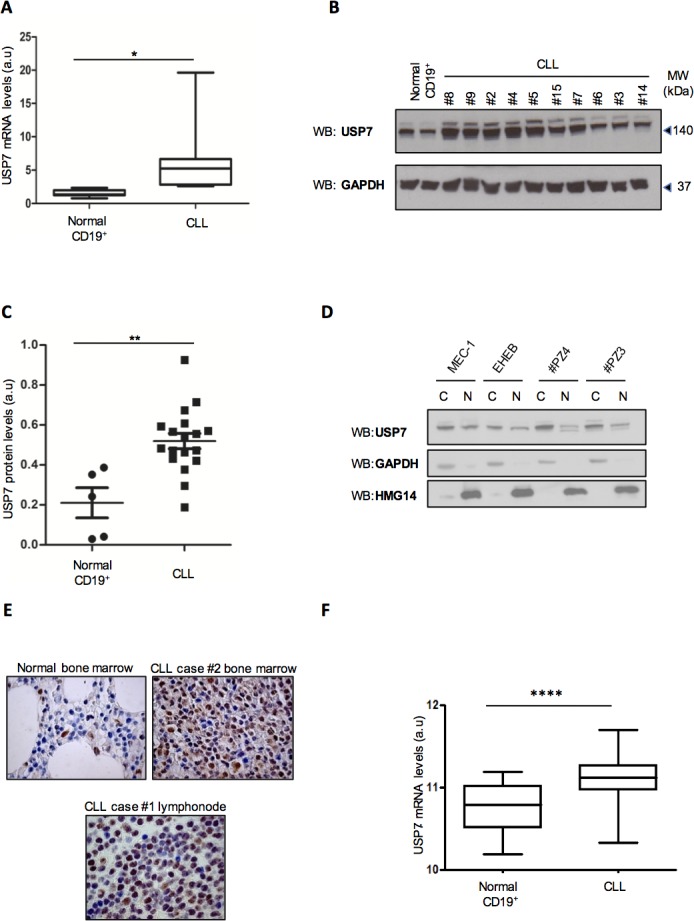
USP7 is strongly up-regulated in CLL samples **A.** Quantification of mRNA levels in 5 normal CD19^+^ lymphocytes and 19 CLL samples. *p < 0.05. **B.** Primary CD19^+^ lymphocytes from two representative normal individuals and ten CLL patients were analyzed for USP7 protein expression. **C.** Quantification of USP7/GAPDH ratio in 5 normal CD19^+^ lymphocytes and 19 CLL samples. **p < 0.01. **D.** Western Immunoblot of cytoplasm/nuclear fractions in CLL cell lines model and two representative primary CLL samples. **E.** USP7 immunohistochemical of human biopsies in one representative normal bone marrow and two CLL specimens. **F.** Box-plot of USP7 mRNA levels in normal lymphocytes (*n* = 12) compared to CLL primary cells (*n* = 217). ****p < 0.0001.

To assess the levels of expression of *USP7* in CLL, real-time PCR was performed on mRNA isolated from primary CD19^+^ lymphocytes of CLL patients and healthy individuals. As reported in Figure 1A, *USP7* mRNA is markedly up-regulated in CLL. Similarly, using protein extracts from primary CD19^+^ lymphocytes of CLL patients and representative healthy individuals, we observed significantly increased levels of USP7 in CLL samples when compared to normal cells (Figure 1B). Most CLL patients showed a USP7/GAPDH ratio higher than normal CD19^+^ lymphocytes, indicating that USP7 was generally over-represented in CLL (Figure 1C). The biological features of enrolled patients were reported in Supplementary Table 1. USP7 is expressed both in the nucleus and in the cytoplasm of representative primary CLL samples and CLL cell lines, MEC-1 and EHEB (Figure 1D), as observed in other cellular models [31–34]. Immunohistochemical analysis showed a strong positivity for USP7 in 3 out of 5 CLL samples when compared to normal lymphocytes in normal bone marrow specimens (Figure 1E). Finally, we analyzed *USP7* expression levels in a publicly available larger cohort of CLL patients (*n* = 217) and 12 normal samples [35]. Also in this case, USP7 was over-expressed in CLL when compared to normal samples (Figure 1F). Although this cohort included only patients with stage A of the Binet classification (i.e. limited-stage disease), USP7 overexpression in CLL is highly significant and therefore these data suggest that its overexpression may represent a common feature even at the early stages of the disease. Altogether these data provide a rationale to investigate USP7 as a target in CLL.

### USP7 is regulated at post-transcriptional and post-translational levels

Prior to investigate USP7 as a potential therapeutic target in CLL, we sought to assess the mechanisms of USP7 overexpression and activation in CLL. Micro-RNAs (miRNAs) have been reported as functional players in CLL pathogenesis with prognostic significance [36]. Therefore, we performed a bioinformatic survey of publicly available datasets [35] returning a list of miRNAs potentially able to target the USP7 3′-UTR (Supplementary Figure S1A). The calculated Pearson correlation coefficient was highly significant for an inverse correlation between USP7 and miR-338-3p and miR-181b (Supplementary Figure S1B and S1C). Thereby, we firstly subcloned the miR-338-3p responsive element (MRE) of *USP7* (Supplementary Figure S1D, upper panel) downstream to a luciferase construct and a reporter assay showed that miR-338-3p could directly down-regulate USP7 at post-transcriptional level (Supplementary Figure S1D, lower panel). Similar data were obtained with miR-181b response element (Supplementary Figure S1E upper and lower panel). Accordingly, miR-338-3p transfection strongly reduced *USP7* levels (Supplementary Figure S1F). We provide the *in vitro* proof of principle that USP7 overexpression in CLL may be sustained through miRNA deregulation, and in particular by miR-338-3p and miR-181b. Interestingly, miR-181b was already shown to be down-regulated in CLL and to play a role in CLL pathogenesis through the regulation of the oncogene Tcl1 [37, 38]. Further analyses are however mandatory to assess the role of miR-338-3p and miR-181b in the setting of larger CLL cohorts, to better correlate their expressions with clinical outcomes and biological characteristics.

Next, we studied USP7 post-translational modifications. In particular, it was already demonstrated that Protein Casein Kinase 2 (CK2) phosphorylates USP7 on serine 18 residue with consequent regulation of protein stability and activation [33].

**Figure 2:**
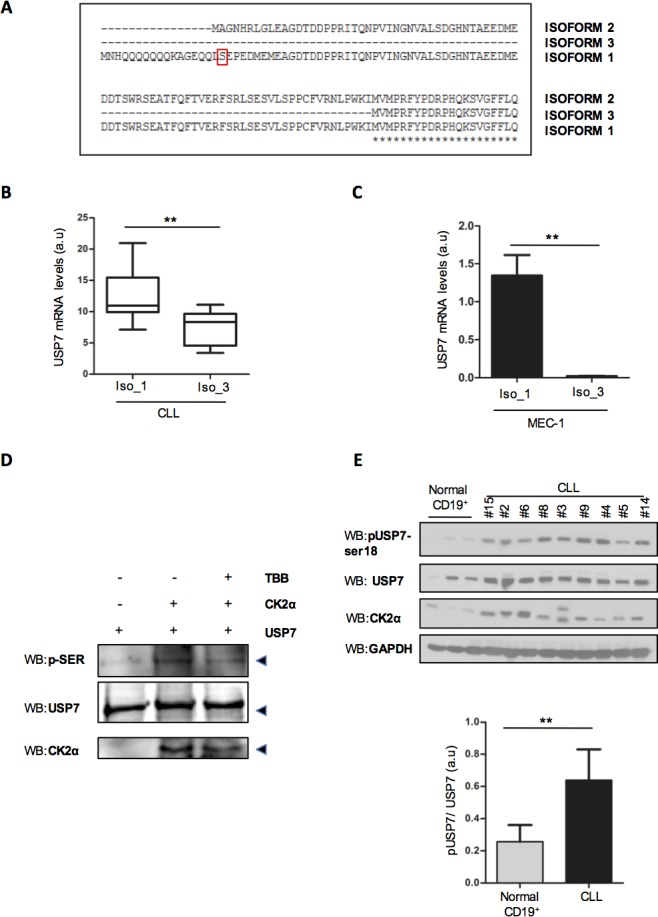
USP7 Post-translational modifications in CLL **A.** Representation of USP7 isoforms segments highlighting the only isoform containing serine-18. **B.** Quantification of USP7 isoform 1 (Iso_1) and isoform 3 (Iso_3) mRNA levels in CD19^+^ lymphocytes from 19 CLL samples. **p < 0.01. **C.** Quantification of USP7 isoform 1 and 3 mRNA levels in MEC-1 cells. **p < 0.01. **D.** Kinase assay with purified USP7 protein and immunoprecipitated CK2. The assay was performed in the absence or presence of 60 μM TBB. p-SER: phospho-serine. **E.** Upper panel: Purified tumor cells from normal CD19^+^ and CLL patients were analyzed for the indicated proteins. Lower panel: quantification of pSer18 USP7 expression level, normalized on total USP7. **p < 0.01.

Despite several USP7 isoforms have been predicted (https://www.ncbi.nlm.nih.gov/gene/7874) (Figure 2A), only USP7 isoform 1 contains serine 18. Due to the relevant biological role of CK2 in CLL pathogenesis [26, 27, 29, 39–43], we sought to investigate whether CK2 is involved in modulating USP7 activity in this disease. We first investigated the levels of USP7 isoforms in primary CLL samples. Unfortunately, we were not able to specifically detect isoform 2. Interestingly, isoform 1 was the most represented variant in CLL primary samples (Figure 2B) and in MEC-1 and EHEB CLL cell lines (Figure 2C and Supplementary Figure S2A). In line with previous results [33], we observed that USP7 is phosphorylated on Ser18 residue in the EHEB and MEC-1 cell lines and phosphorylation was reverted by CK2-inhibitor treatment (Supplementary Figure S2B). Notably, CK2 inhibitor treatment did not affect total USP7 mRNA levels (Supplementary Figure S2C) but, as observed elsewhere [33], a prolonged treatment with 25 μM TBB for 72 hours was associated with USP7 protein reduction (Supplementary Figure S2D). Since these cells expressed mostly USP7 isoform 1, the one with serine 18, these data suggest that CK2 promotes USP7 stabilization in CLL context, through phosphorylation. On the contrary, CK2 inhibitors did not chance the localization of USP7 (Supplementary Figure S2E). A kinase assay of purified USP7 incubated in the presence of CK2 confirmed that CK2 phosphorylates USP7 (Figure 2D). Finally, also phospho-Serine-18 specific antibody demonstrates that USP7 is highly phosphorylated on this residue in primary CLL samples (Figure 2E). Overall, our data show that USP7 overexpression may be correlated to miRNAs deregulation and that USP7 is functionally under the upstream control of CK2, thus enforcing the functional role of USP7 in CLL.

### Targeting USP7 induces growth inhibition and apoptosis in CLL cell lines

**Figure 3:**
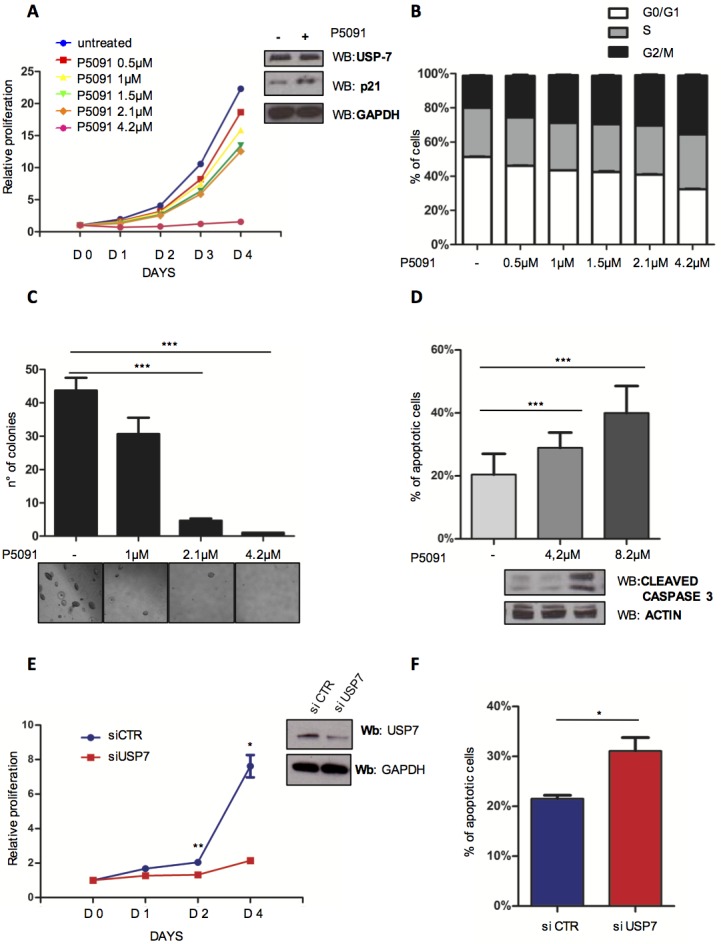
Targeting USP7 induces growth inhibition and apoptosis in CLL cell lines **A.** Proliferation analysis of MEC-1 cells treated with USP7 inhibitor (P5091) for the indicated times and concentrations. The number of cells at day 0 was set at 1 unit. Insert: lysates from control and P5091-treated MEC-1 cells were analyzed for p21 expression level. **B.** Cell cycle distribution of MEC-1 cells treated or not with P5091 for 3 days. **C.** Quantification and representative images of soft-agar growth assay in MEC-1 cells treated with the indicated concentrations of P5091 for 15 days. ***p < 0.001. **D.** Upper panel: evaluation of apoptosis in MEC-1 cells treated with the indicated concentrations of P5091 for 24 hrs. ***p < 0.001. Lower panel: cleaved-caspase-3 Western Immunoblot in the same conditions. **E.** Proliferation analysis in MEC-1 cells infected either with control (siCTR) or USP7-directed (siUSP7) siRNAs (insert). *p < 0.05; **p < 0.01. **F.** Apoptosis analysis of MEC-1 cells described in E. *p < 0.05.

Recently a few inhibitors have been developed to specifically target USP7 with promising results in different cancer models [17, 44–50]. USP7 inhibitor P5091 was shown to exhibit impressive properties for the treatment of Multiple Myeloma (MM) [17]. To explore the potential therapeutic role of USP7 inhibition in CLL, we treated the *TP53*-mutated CLL cell line MEC-1 with P5091. P5091 treatment was associated with: i) dose-dependent reduction of cell viability *via* p21 at a concentration as low as 1 μM (Figure 3A, left panel and insert) through MDM2 (Supplementary Figure S3A and S3B), with a similar mechanism as described previously [17]; ii) accumulation in the G2/M phase of the cell cycle (Figure 3B); iii) inhibition of anchorage-independent growth (Figure 3C, upper panel and lower panel) and iv) activation of the apoptotic pathway as indicated by the proteolytic cleavage of Caspase 3 (Figure 3D upper and lower panel). Similar results were obtained also in the *TP53*-wild type EHEB CLL cell line (Supplementary Figure S4A and S4B). Interestingly, both *TP53*-mutated MEC-1 and *TP53* wild-type EHEB displayed a similar P5091 IC_50_ (MEC-1 1.3 μM; EHEB 2 μM), implying a similar p53-independent “response phenotype” (Supplementary Figure S4C). Notably, despite being characterized by *TP53* deletion, MEC-1 cells were highly responsive to P5091, suggesting that USP7 inhibition can overcome *TP53* deletion-related resistance to apoptosis. P5091 treatment did not affect p53 targets in MEC1 cell line (Supplementary Figure S5A) and PTEN levels did not change (Supplementary Figure S5B).

To verify the specificity of the USP7 inhibitor, we then used a pool of 5 different siRNAs able to efficiently silence USP7 (Supplementary Figure S6A). USP7 silencing in MEC-1 cells (Figure 3E and Supplementary Figure S6B) abrogated cell growth, corroborating the *bona fide* data we obtained with the inhibitor. Consistently, USP7 silencing promoted MEC-1 apoptosis (Figure 3F). Collectively, these data demonstrate that USP7 targeting through small molecule inhibition is highly effective in CLL cell lines.

Lastly, we assessed whether P5091 treatment synergized with one of the recently approved drugs, idelalisib [15, 51]. We performed proliferation and apoptosis assays using MEC-1 and EHEB cell lines. Interestingly, combined treatment of MEC-1 and EHEB cell lines with the PI3K-delta and USP7 inhibitors synergistically inhibits proliferation and promotes apoptosis induction (Supplementary Figure S7A-S7F). These results suggest that USP7 inhibition may impact on pathways that may be PI3K/AKT independent.

### USP7 inhibition affects PTEN delocalization

**Figure 4:**
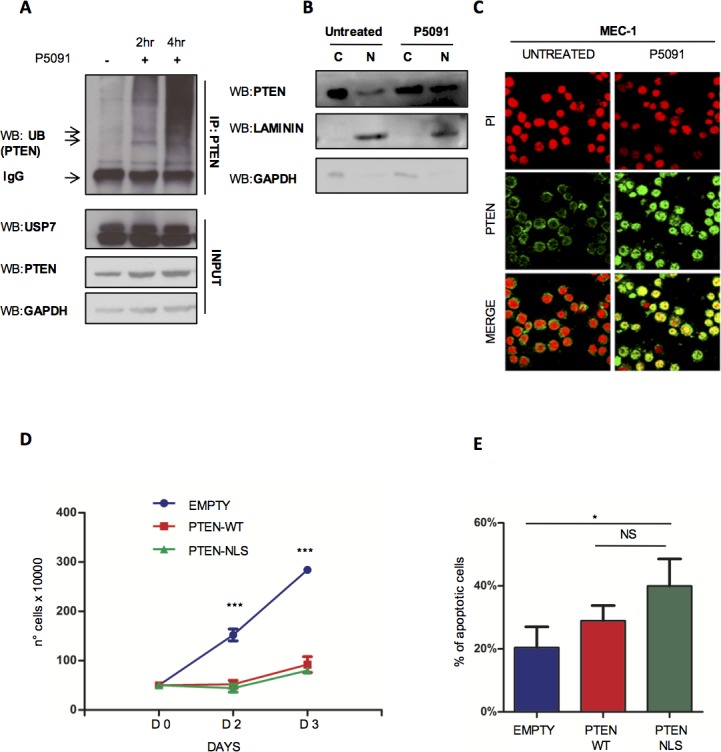
USP7 inhibition affects PTEN delocalization **A.** PTEN-ubiquitination assay in MEC-1 cells treated with 16 μM P5091 for 2 and 4 hours. **B.** USP7 Western Immunoblot upon cytosol/nuclear fractionation of MEC-1 cells treated with P5091 at a concentration of 16 μM for 2 and 4 hours. **C.** Immunofluorescence assay showing PTEN cellular compartmentalization in MEC-1 cells treated with 4.2 μM P5091 for 24 hours. Anti-PTEN antibody (green); Propidium iodide (red). **D.** Proliferation analysis of MEC-1 cells expressing GFP-PTEN-WT and GFP-PTEN-NLS. Cell growth was quantified by counting cells with trypan blue exclusion of dead cells at each time point. ***p < 0.001. **E.** Percentage of apoptosis in GFP-PTEN transfected MEC-1 cells. *p < 0.05.

USP7 is a de-ubiquitinase that targets three major tumor suppressors: p53 [18], PTEN [19, 20] and FoxOs [52]. USP7 promotes the deubiquitination of: i) p53, increasing its stability *via* MDM2; ii) PTEN, favoring its shuttling from the nucleus into the cytoplasm; iii) FOXOs, favoring changes in cellular compartmentalization. By changing tumor suppressors' cellular compartmentalization and protein levels, USP7 plays an essential and direct role in tumorigenesis, while its inhibition offers the challenging therapeutic options to reactivate tumor suppressive functions and trigger cancer selective apoptotic response. Our observation that P5091 markedly promoted apoptosis in *TP53*-mutated MEC-1 cells suggests that P5091 effects are p53-independent in CLL. Since we previously demonstrated that USP7 plays an essential role in the regulation of PTEN compartmentalization in CML [20, 34], we sought to investigate whether USP7 functionally inactivates PTEN in CLL through PTEN nuclear exclusion. PTEN plays a role in CLL pathogenesis where it is not mutated/deleted [25] but functionally inhibited. Therefore, we speculated that USP7 inhibition may promote PTEN reactivation and consequently cancer selective apoptotic induction, without affecting normal cells. PTEN is an essential tumor suppressor implicated in tumorigenesis through either phosphatase dependent and independent functions [53]. In particular, it is involved in the negative regulation of the PI3K-AKT signaling, a major pathway responsible to CLL maintenance, and acts in the nucleus where it regulates proliferation [54] and genomic stability [55, 56]. As shown in Figure 4A, treatment with USP7 inhibitor restored PTEN endogenous ubiquitination levels. Therefore, P5091 promoted PTEN re-localization into the nucleus, as detected by cell fractionation (Figure 4B) and immunofluorescence (Figure 4C) of MEC-1 cell line, but did not changed PTEN protein levels (Supplementary Figure S5B). Also, the inhibition of the USP7 upstream regulator CK2 with TBB promoted PTEN shuttling, therefore indicating that CK2/USP7/PTEN forms an important network in CLL cells (Supplementary Figure S8A). To confirm the tumor suppressive role of nuclear PTEN in CLL cells, we forced PTEN expression into the nucleus upon transfection with a Nuclear Localization Signal (NLS)-bearing PTEN construct. Nuclear PTEN expression was associated with growth arrest (Figure 4D) and apoptosis induction (Figure 4E), suggesting that PTEN acts as a potent tumor suppressor in CLL. Our data demonstrate that USP7 is responsible for PTEN nuclear exclusion with consequent impairment of its tumor suppressive functions and that USP7 inhibition restores PTEN nuclear pool and its oncosuppressive activity in the context of CLL.

### P5091 affects USP7/PTEN network in primary CLL samples

**Figure 5:**
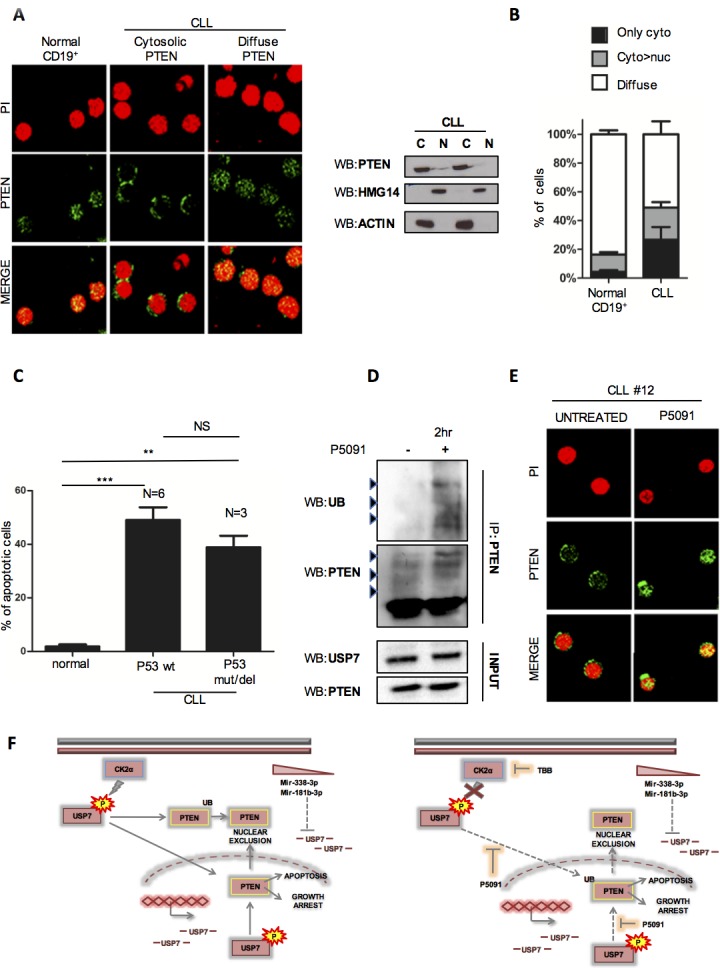
P5091 affects USP7/PTEN network also in primary CLL samples **A.** Left panel: Representative immunofluorescence images on CD19^+^ lymphocytes from peripheral blood of normal and CLL individuals, stained with anti-PTEN antibody (green) and Propidium iodide (red). Right panel: Western Immunoblot of cytoplasm/nuclear fractions in two representative primary CLL samples. **B.** Quantification of PTEN cellular compartmentalization in CD19^+^ lymphocytes from 5 normal and 14 CLL samples. Data represent mean ±SD of > 50 cells analyzed from each sample and expressed as percentage. **C.** Apoptosis analysis of CD19^+^ lymphocytes from normal (*n* = 3) and CLL (*n* = 9) individuals treated with P5091 for 24 hours. Percentage of each sample was normalized to corresponding untreated condition. ***p < 0.001. **D.** PTEN ubiquitylation assay in CLL primary cells treated with DMSO or P5091 (16 μM) for 2 hours. **E.** PTEN cellular compartmentalization (green) in a representative PTEN nuclear-excluded CLL sample before and after treatment with P5091 (4.2 μM) for 24 hours. **F.** Model of USP7 network in CLL.

We evaluated PTEN cellular compartmentalization in primary CLL samples by immunofluorescence and western immunoblot upon nuclear/cytoplasmic fractionation. Interestingly, normal CD19^+^ lymphocytes displayed a physiological localization of PTEN both in the nucleus and the cytosol (which we refer to as a “diffuse pattern”), while most of CLL CD19^+^ lymphocytes were characterized by PTEN nuclear exclusion (from an exclusively “cytosolic” PTEN to a “predominantly cytosolic” compartmentalization of PTEN) (Figure 5A and Supplementary Figure S9A). Although few cells in the control group may display cytosolic expression of PTEN, a fact recalling a physiological degree of dynamic regulation, the overall statistics across CLL lymphocytes clearly showed a major unbalance of the PTEN pool toward the cytoplasm (Figure 5B). This latter result confirms the relevance of these findings in human disease. To corroborate the results in CLL cells lines, we decided to test the impact of P5091 in CLL samples. Also in primary cells, P5091 treatment strongly promoted apoptosis (Figure 5C and Supplementary Figure S9B) while B-lymphocytes from normal individuals were unaffected. Only higher concentrations of P5091 (16 μM) decrease the viability of normal lymphocytes (Supplementary Figure S9C), suggesting a potential selectivity of the therapy. Strikingly, three *TP53* deleted CLL samples were equally subjected to P5091 apoptosis induction, confirming that USP7 inhibitor acts in a p53-independent manner. Moreover, USP7 inhibitor treatment of primary CLL was associated with increased ubiquitination of endogenous PTEN and consequently PTEN re-localization into the nucleus (Figure 5D and 5E). These data demonstrated a potent activity of P5091 against primary CLL cells.

## DISCUSSION

Several effective drugs for the treatment of CLL have been recently licensed while others are in advanced phases of clinical development. Notwithstanding, the effective treatment of *TP53* mutated or deleted (del17p) CLL patients remains challenging. *TP53* mutations/deletions are involved in CLL transformation into a more aggressive disease (i.e. Richter syndrome) and may reside in dormant clones in the very early stages of the disease [57]. Indeed, treatments exert a selective pressure favoring the expansion of *TP53*-mutated/deleted clones; despite *TP53* restoration in tumors affects cancer maintenance, no specific therapies have been developed to effectively overcome *TP53* mutated tumors.

Overall, in this study we utilized CLL cell lines and primary tumor cells as models to investigate the USP7 role in CLL and to demonstrate the efficacy of a small molecule inhibitor P5091, in this disease. As summarized in Figure 5F, our data show that the de-ubiquitinase USP7 is aberrantly expressed in CLL. USP7 is over-expressed in about 70% of CLL CD19^+^ lymphocytes, both at the mRNA and protein levels. This observation has been originally made using our (single) institute cohort of CLL patients and extended within a previously published mRNA expression dataset counting 217 CLL cases [35]. It should be questioned that this cohort included only patients in the Binet A CLL stage, which represents the early stage of the disease. However, it should be considered that in other cancer types USP7 over-expression has been commonly correlated with both disease progression and advanced stages, therefore suggesting that USP7 over-expression in CLL may play a pathogenetic role even in the early stages of this disease.

Following identification of USP7 aberrant expression in CLL primary cells, we investigated the mechanisms responsible to its deregulation. High USP7 expression appeared to be a consequence of deregulated miRNA expression and/or post-translational regulation by CK2, an aberrantly expressed serine-kinase in CLL. miRNA expression profiling revealed that miR-338-3p and miR-181b anticorrelate with USP7 in CLL samples and *in vitro* experiments confirmed their direct interaction with the 3′UTR of the gene. Unluckily, due to the inability to demonstrate miR-338-3p down-expression in CLL samples *versus* normal individuals in this dataset, its real role in CLL pathogenesis requires further investigations. It could be speculated that the contribution of miR-338-3p in USP7 regulation is different in CLL respect to normal lymphocytes or that miR-338-3p is involved in CLL pathogenesis through different mechanisms. Conversely, miR-181b is already known to be downregulated in CLL and to play a key role in CLL pathogenesis [37, 38]. While further analyses are mandatory to dissect the role of these miRNAs in the regulation of USP7 in CLL, our data clearly confirmed that CK2-mediated USP7 phosphorylation enhances its de-ubiquitinase activity in CLL. More precisely, we found that CLL samples mostly expressed the serine-18 USP7 isoform and that CK2-mediated phosphorylation plays a key role in the regulation of USP7 activity and consequently of its targets, as demonstrated elsewhere [33]. While further analyses should be performed to better dissect the CK2/USP7/PTEN network, due to the ability of CK2 to activate USP7 as a deubiquitinase and to directly promote PTEN tail phosphorylation, these findings provided the rational for the evaluation of USP7 inhibitor in CLL and therefore we focused on anti-tumor activity of its inhibitor, P5091. We showed that treatment with low concentrations of the USP7 inhibitor selectively induces apoptosis of MEC-1 and EHEB CLL cell lines and primary CLL CD19^+^ lymphocytes, while maintaining unaffected CD19^+^ lymphocytes from normal individuals. P5091 treatment effectively induces apoptosis in both *TP53*-mutated MEC-1 and *TP53*-wild-type EHEB cell lines and, remarkably, is highly effective in del17p CLL primary CD19^+^ lymphocytes, with an apoptotic induction ‘potency’ like the one observed in *TP53*-wild type CLL samples.

To better evaluate the mechanisms of USP7 anti-leukemic properties in *TP53*-wild-type and mutated CLL, we then analyzed USP7 targets in this specific context. USP7 was originally studied as a gene able to regulate p53 through a complex MDM2/p53 network. It also targets two major tumor suppressors: PTEN and FoxO(s). Specifically, USP7 induces the de-(mono)-ubiquitination of PTEN and FoxO, promoting their nuclear exclusion; on the contrary, mono-ubiquitination favors an enrichment of the nuclear pool. Nuclear PTEN targets cell cycle [54] and the machinery that regulates genomic stability [55, 56], thus protecting genomic integrity. PTEN was described as a functionally inactive tumor suppressor in CLL. Notably, no genetic alterations of PTEN have been reported in CLL, suggesting that therapies that promote PTEN reactivation may effectively target CLL cells.

In line with these considerations, we have here observed that USP7 promotes PTEN delocalization in CLL samples, favoring the loss of its nuclear tumor suppressive functions. Conversely, USP7 inhibition is associated with the restoration of the PTEN nuclear pool and correlates with apoptosis induction and cell growth arrest. The functional reactivation of tumor suppressor PTEN can indeed by-pass the apoptotic resistance of *TP53* mutated cells/clones.

While USP7 involvement in tumorigenesis should be further investigated (e.g. in deep studying USP7 targeting miRNA(s) and regulation of CK2 enzymatic activity), our data attribute to USP7 inhibitors a therapeutic role in CLL precision medicine, functioning through the reactivation of the tumor suppressor PTEN. In doing that, USP7 inhibitor P5091 activates an apoptotic and cell growth arrest response which occurs by-passing *TP53* genetic loss.

## MATERIALS AND METHODS

### Cells and primary human samples

Human samples were collected from *n* = 20 untreated CLL patients at the San Luigi Hospital (Orbassano, Italy), following informed consent and with obscured identity; CD19+ lymphocytes were isolated from healthy donors (*n* = 5) accordingly to the MiltenyiBiotec protocol (MiltenyiBiotec, #130-050-301). The project was reviewed and approved by the institutional ethical committee (code #10/2013). Human MEC-1 cell line was gently provided by Prof. Deaglio (University of Turin) and was grown in IMDM medium (Life Technologies) containing 10% heat-inactivated fetal bovine serum (FBS, Sigma Aldrich), supplemented with 2 mM L-glutamine, 100 U/ml penicillin, and 0.1 mg/ml streptomycin and maintained at 37°C in a 5% CO2 humidified atmosphere. EHEB cell line was purchased from DSMZ and was maintained in RPMI 10% FBS. The lymphoid nature of this cell line was authenticated by flow-cytometry (CD19^+^ positivity).

*TP53* status of MEC-1 and EHEB was previously investigated [58]. HEK293T cells were obtained from ATCC and were maintained in DMEM supplemented with 10% FBS. Cell lines were regularly tested with MycoAlert (Lonza) to ascertain that cells were not infected with mycoplasma.

### Plasmids, silencing and nucleofection

GFP and PTEN constructs were described elsewhere [20, 59]. Specific USP7 siRNA was purchased from Sigma-Aldrich (EHU-1311171). Negative Control siRNA from Ambion (Cat#4611G) was used as a control. For the transient transfection 1.5 × 10^6^ MEC-1 cells were pelleted and resuspended in 100μl of Nucleofector solution (Lonza), according to the manufacturer's protocol. For each nucleofection, 2 μg of plasmid vectors or 30 nM of siRNAs were placed in the cuvette. pMaxGFP® (Lonza) was used to evaluate transfection efficiency. Nucleofection was done using program V-001 or U-015 on the Shuttle System. After nucleofection, the content of each cuvette was dispersed as rapidly as possible with 500 μl of the pre-equilibrated culture medium and transferred into a 12-wells plate. Twenty-four hours after transfection experiments were performed.

### Luciferase reporter assays

100 ng of luciferase plasmids (psiCHECKTM-2) encoding wild-type or mutant 3′UTR of USP7 were co-transfected with synthetic pre-miRNA (hsa-miR-338-3p, AMBIONPM10716) or pre-miRNA (has-miR-181b-3p, AMBIONPM20327) in HEK 293T cells with Lipofectamine 2000 (Invitrogen) and the luciferase assay was performed with the Dual-Luciferase Reporter Assay System (Promega). Luminescence was measured with the “Dual Glow” protocol of the GloMax MULTI Detection System (Promega). The luciferase signal obtained from Renilla was normalized against Firefly luciferase intraplasmid control. The USP7 sensor vector (containing the single miR-338-3p and miR-181b MRE) was obtained by annealing the following oligonucleotides:

USP7SENSOR_WT_338-3p_FW: tcgagCCTGTATATTGCCTTTTTGCTGGAAAAaagcttgc

USP7SENSOR_WT_338-3p_RV:

ggcc gcaagcttTTTTCCAGCAAAAAGGCAATATA CAGGc

USP7SENSOR_WT_181b_FW:

tcgagTATAAAAATTATAATTCAGTGAGcatatggc

USP7SENSOR_WT_181b_RV:

ggccgccatatgCTCACTGAATTATAATTTTTATAc

The same procedure was used to generate the mutated sensor using the following oligonucleotides:

USP7SENSOR_MUT_338-3p_FW:

tcgagCCTGTATATTGCCTTTTCTAC TTCAAAaagcttgc

USP7SENSOR_MUT_338-3p_RV:

gg ccgcaagcttTTTGAAGTAGAAAAGGCA ATATACAGGc

USP7SENSOR_MUT_181b_FW:

tcgagTATAAAAATTATAATGACTGTCGcatatggc

USP7SENSOR_MUT_181b_RV:

ggccgccatatgCGACAGTCATTATAATTTTTATAc

### Antibodies and inhibitors

PTEN (#9188S), Phospho-Serine (#9631S), GAPDH (#5174), Cleaved Caspase-3 (#9661), NOXA (#14766), BAX (#2772), PUMA (#4976) and HSP90 (#4874) were from Cell Signaling Technologies; PTEN (#sc133197), CK2 (#sc12738), Laminin (#sc-6216), USP7 (#sc30164), P21(#sc817), MDM2 (#sc965) were from Santa Cruz; Anti-Ubiquitin was from BD Pharmigen (#550944); GFP (A6455) was from Invitrogen; Tubulin (#T5201), Actin (#A5060) were from Sigma-Aldrich. HMG14 was from Abcam (#ab5212); pSer18 USP7 (#ABC225) was from Millipore. USP7 inhibitor (P5091), Idelalisib (CAL-101, GS-1101) and CK2 inhibitor (TBB) were from Selleckchem and Sigma-Aldrich, respectively.

### Cell lysis, western blot assay and immunoprecipitation

For cytosol/nuclear fractionation cell pellets were resuspended in hypotonic lysis buffer (10 mM Hepes pH 7.9, 10 mM KCl, 0.1 mM EDTA pH 8.0, protease inhibitors and phosphatase inhibitors) and incubated on ice for 20 minutes. After the addition of NP40, samples were centrifuged at 1200×g for 5 minutes and cytosolic extracts were recovered. Nuclear pellets were resuspended in nuclear extraction buffer (20 mM Hepes pH 7.9, 0.4M NaCl, 1 mM EDTA pH 8.0, protease and phosphatase inhibitors). The lysates were subjected to alternative vortex mixing and ice-cooling, and after centrifugation the nuclear extracts were collected. Immunoprecipitation was performed using whole-cell lysates. Proteins were extracted with lysis buffer containing 150 mM NaCl, 1 mM EDTA, 50 mM Hepes (pH 7.5), 1% Triton X-100 and 10% glycerol. Then lysates were incubated with antibodies overnight at 4°C on a rotator. Protein A/G-PLUS-Agarose beads (Santa Cruz #2003) were added and incubated for 2 hours at 4°C with rotation. Beads bound with immunoreactive complexes were washed four times with cold lysis buffer. Immunoprecipitated complexes were boiled for 5 min and subsequently ran using SDS-PAGE on 8% or 6% gels and transferred onto nitrocellulose filters. Immunoblots were then probed overnight at 4°C with specific antibodies in PBS-0,1% tween-1% BSA and protein detection was performed by using peroxidase-conjugated secondary antibodies and chemiluminescence reagent (BIORAD, #170-5060). A similar protein running and transfer procedure was used for Western blotting.

### Kinase assay

*In vitro* kinase assay was performed with immunoprecipitated CK2 kinase and purified His-GST-USP7 (Invitrogen, #11681-H20BL-50), in basal conditions or in presence of CK2 inhibitor TBB (60 μM). After 30 minutes at 37°C, reaction was stopped at 95°C and Western Blot was performed.

### Gene expression analysis

RNA was extracted from cells using TRIzol (Invitrogen). 1 μg of total RNA was used for reverse transcription. Real-time PCR was performed with iQ SYBR Green (Bio-Rad). Primers used were:

hUSP7_TOT_FW: 5′-CGCTGGGGAACATGGCTTAC-3′

hUSP7_TOT_RV: 5′-TTGGTCCGTCTGAGGGTCAT-3′

hUSP7_ISO1_FW:5′-CCGAGGACATGGAGATG GAAG-3′

hUSP7_ISO1_RV: 5′-CGCCAACTGGTGTCAT CCTC-3′

hUSP7_ISO3_FW:5′-TGCCAAAAGTTCAGCCT CCAT-3′

hUSP7_ISO3_RV: 5′-GGCTAAGGACCGACT CACTCA-3′

hHuPO FW: 5′-GCTTCCTGGAGGGTGTCC-3′

hHuPO RV: 5′-GGACTCGTTTGTACCCGTTG-3′

Real-time PCR parameters were: cycle 1, 95°C-3 minutes; cycle 2, 95°C-15 seconds, 60°C-30 seconds for 40 cycles. For p21 quantification specific assays with on-demand primer/probe kits (Hs00355782_m1 for p21) (Applied Biosystems, Foster City, CA, USA) were conducted according to the manufacturer's instructions. The 2-ΔΔCT method was used to analyze the data. hHuPO was used to normalize the results.

### Gene and miRNA expression data analysis

Expression analysis of *USP7* was assessed in a panel of 217 CLL relative to 12 control samples available at GEO (GSE51528) [35]. Samples were assayed on Affymetrix Human Gene 1.0 ST and have been normalized by the RMA algorithm. Differential Expression analysis has been performed using GEO2R tool. The correlation between the gene USP7 and its putative targeting miRNAs was evaluated on 210 matching samples. miRNAs were assayed on Agilent-019118 Human miRNA Microarray 2.0 G4470B available at GEO website (GSE51527) [35]. A log2 transformation has been applied to the miRNAs expression levels. The Pearson coefficient has been used to evaluate the correlation between the USP7 gene and miR-338-3p or miR181b.

### Immunofluorescence and immunohistochemistry

Immunofluorescence was performed as previously described [20]. Immunohistochemistry experiments were performed on formalin-fixed, paraffin-embedded tissues from *n* = 5 patients, as previously reported [60].

### Cell proliferation assay, cell-cycle analysis and assessment of apoptosis

For proliferation, cells were seeded in 96-well plates at a density of 1.5×10^3^ cells/well. Proliferation was evaluated by CellTiter-Glo (Promega) following the manufacturer's instructions. Cell-cycle analysis and apoptosis were performed as previously described [61].

### Anchorage-independent cell-growth assay

Cells were suspended in 0.45% type VII low-melting agarose in 10% IMDM at a density of 5×10^3^ cells/well and plated on a layer of 0.9% type VII low-melting agarose in 10% IMDM in 6-well plates, then cultured at 37°C with 5% CO_2_. After 2 weeks, colonies were counted and images were acquired at 5x magnification.

### Statistical analysis

Two-sided Student's *t-*test or two-way ANOVA with Bonferroni post-test were calculated using GraphPad Prism v5.0d (GraphPad Software). *P*-values < 0.05 were considered statistically significant. *P < 0.05; **P < 0.01; ***P < 0.001; ****P < 0.0001. All mean values (± SEM) are from 3 independent experiments.

## SUPPLEMENTARY MATERIALS FIGURES AND TABLE




