# Supplementary material for: Therapeutic inhibition of USP7-PTEN network in chronic lymphocytic leukemia: a strategy to overcome TP53 mutated/deleted clones
Source: Oncotarget. 2017 Mar 17;8(22):35508–22. doi: 10.18632/oncotarget.16348 (PMC5482594; doi:10.18632/oncotarget.16348)
Supplement: Supplementary file 1 [file oncotarget-08-35508-s001.pdf]

Therapeutic inhibition of USP7-PTEN network in chronic lymphocytic leukemia: a strategy to overcome *TP53* mutated/deleted clones

Supplementary Materials

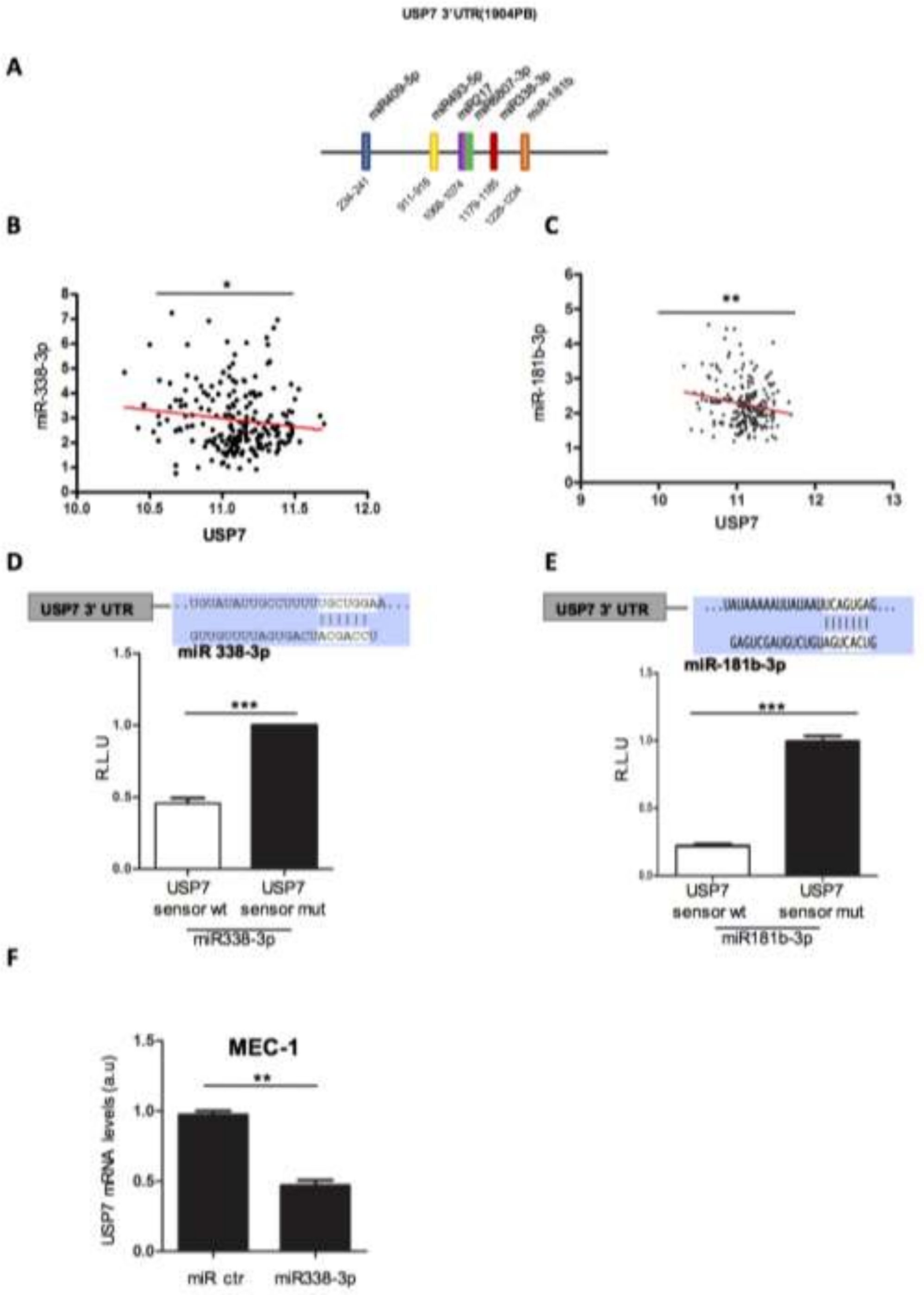

Supplementary Figure S1: miR-338-3p and miR181b-3p directly target USP7. A. Schematic representation of MREs positions in the USP7 3'UTR. **B.-C.** Correlation

between USP7 and its miRNAs, analyzed in a cohort 210 samples. Axes indicate log2 expression data. **D.-E** Upper panel: representative scheme of miR338-3p and miR181b-3p promoters, indicating the positions of oligos utilized in Luciferase assays. Lower panel: relative luciferase expression of miR-338-3p and miR181b-3p promoters sensor constructs (wild type or mutated) in HEK293T cells. ***p<0.001. **F.** Quantification of USP7 mRNA levels after nucleofection with pre-miR 338-3p in MEC-1 cell line. **p<0.01.

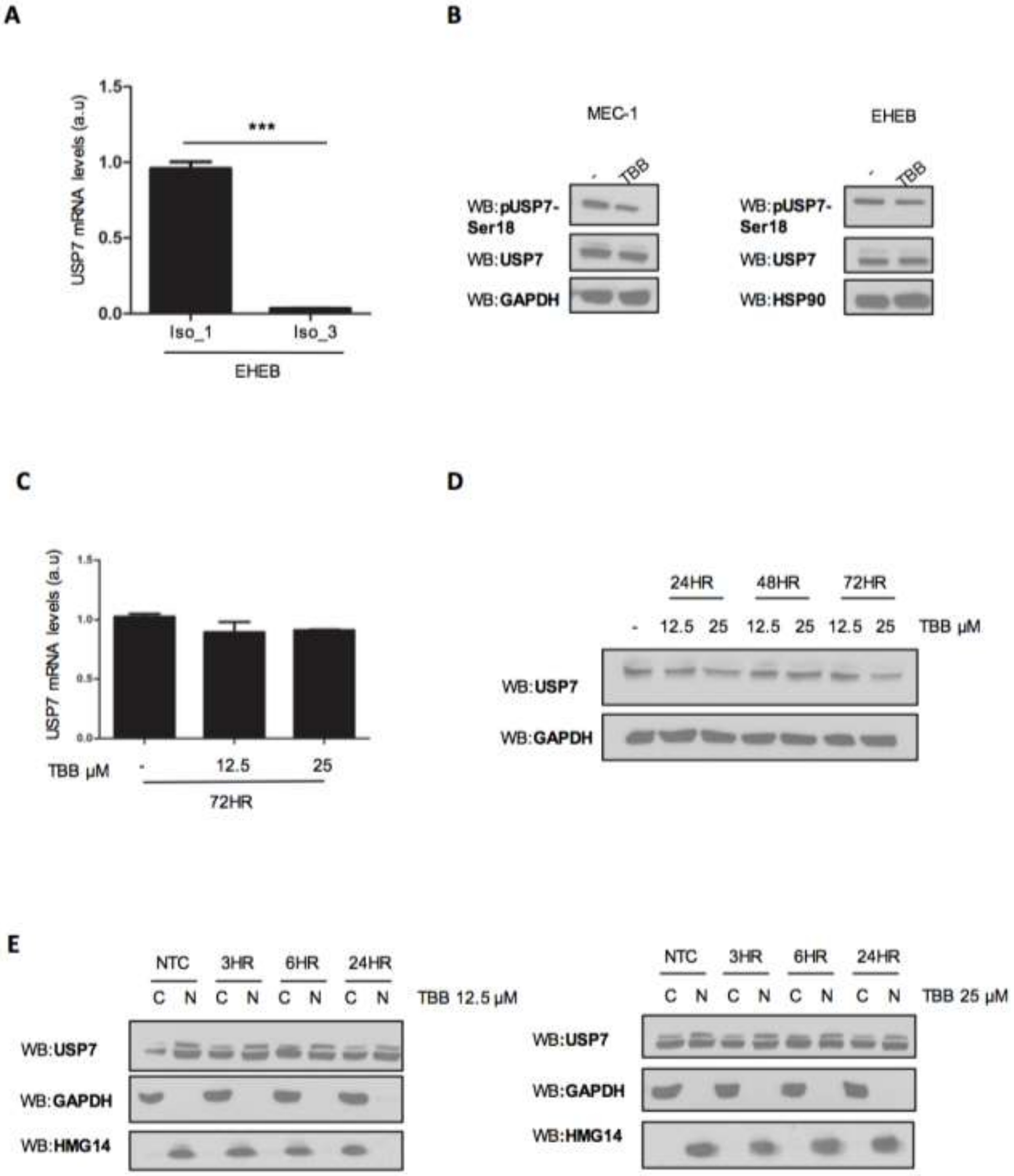

Supplementary Figure S2: USP7 is phosphorylated by CK2 at serine-18 residue. A. Real-time PCR analysis of the indicated in EHEB cell line. ***p<0.001. **B.** EHEB and MEC-1 cells were treated with DMSO or with TBB 25 µM for 24 hours; protein lysates were

analyzed with pSer18 USP7 antibodies. **C.** Real-time PCR analysis of USP7 mRNA levels after treatment with TBB in MEC-1 cell lines. **D. - E.** MEC-1 cells were treated with DMSO or with TBB; protein lysates were analyzed with USP7 antibodies.

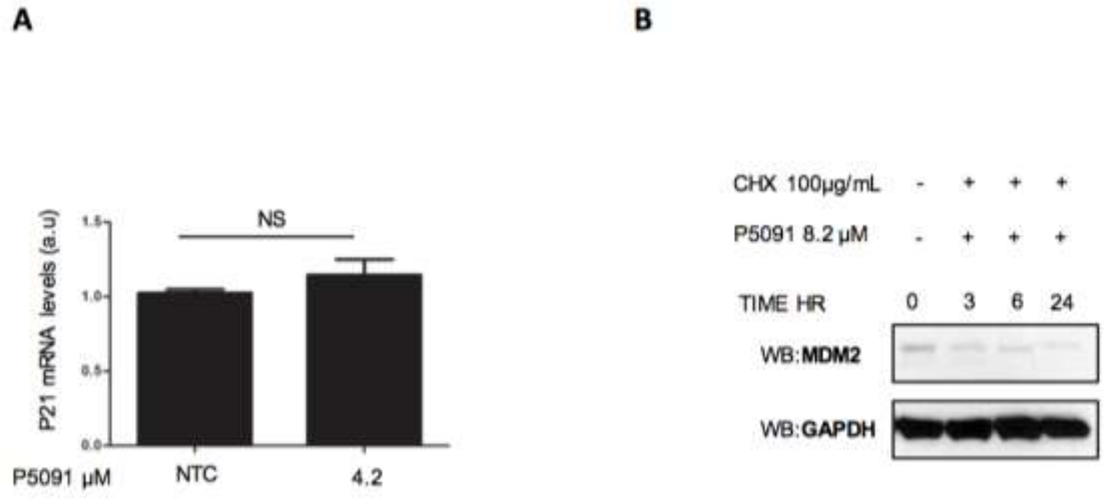

Supplementary Figure S3: p21 and Mdm2 expression regulation in MEC-1 cell line.

A. Real-time PCR analysis of p21 mRNA levels after treatment with P5091 for 24 hours in MEC-1 cell lines. **B.** MEC-1 cells were pretreated with P5091 (8.2 µM,) for 3, 6 and 24 hours, followed by addition of CHX (100 µg/ml) for the indicated times. Total proteins lysates were subjected to immunoblotting with anti-MDM2 or anti-GAPDH antibodies.

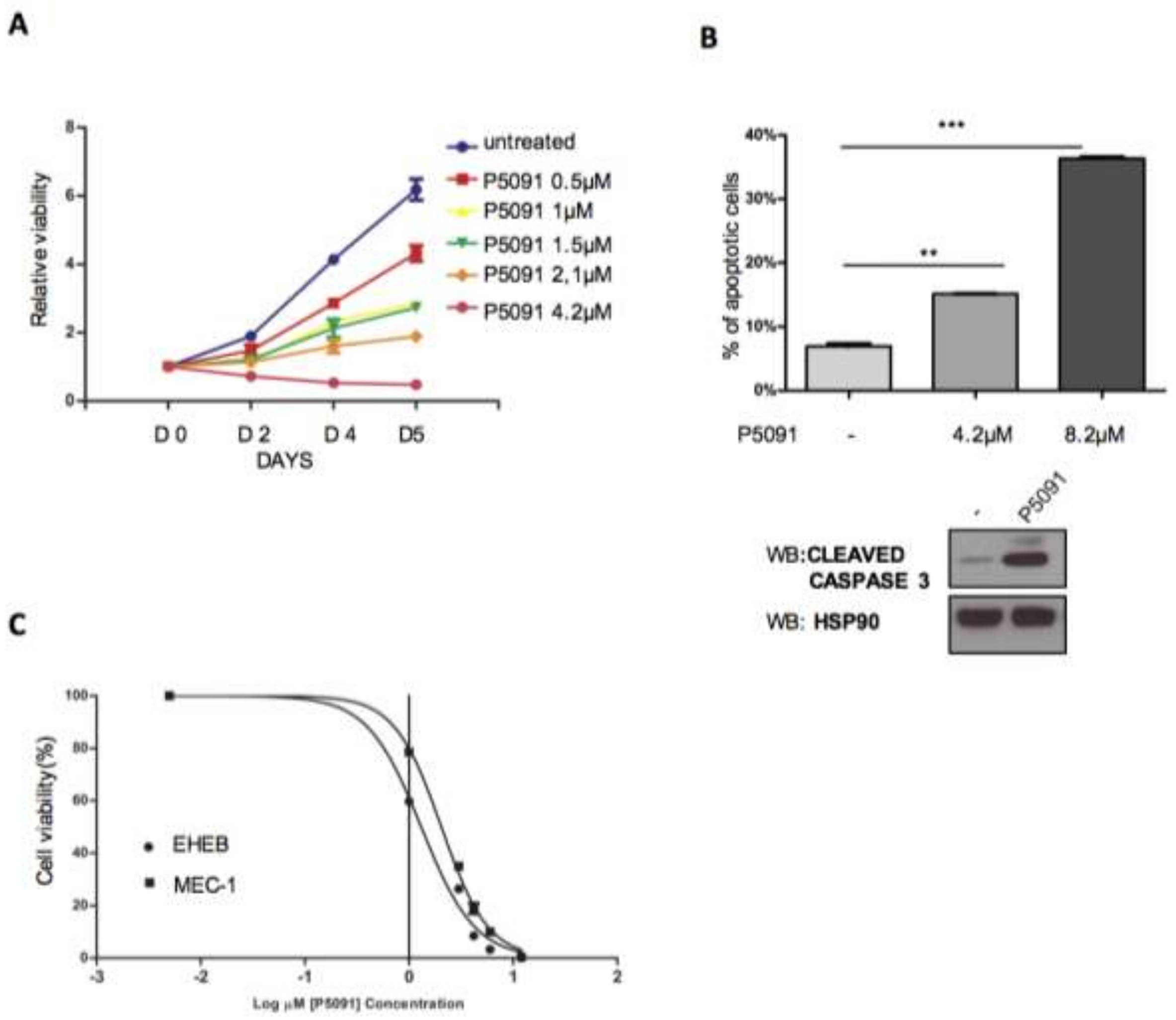

Supplementary Figure S4: P5091 induces apoptosis and growth arrest in EHEB p53-WT cell line. A. Proliferation analysis of EHEB cells treated with USP7 inhibitor (P5091) for the indicated times and concentrations. The number of cells at day 0 was set at 1. Representative experiment of three replicates. **B.** Upper panel: apoptosis assessment of EHEB cells treated with the indicated concentrations of P5091 for 24 hours. $^{**}p<0.01$; $^{***}p<0.001$. Lower panel: representative Western Immunoblot to detect Caspase-3 Cleavage. **C.** Dose-response curves of MEC-1 and EHEB cells lines treated for 48 hours with different P5091 concentrations (0.005-1-3-4.2-6.2-12µM) analyzed by CTG assay (mean r^2=0.99).

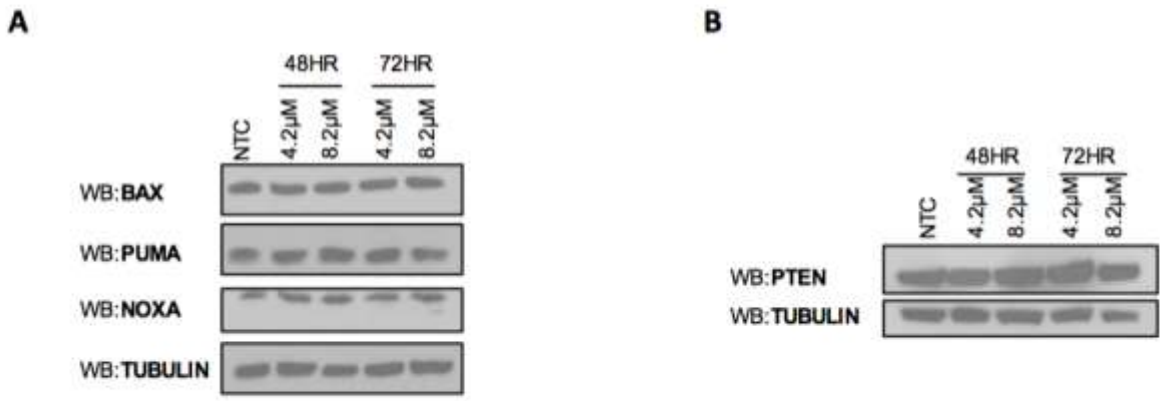

Supplementary Figure S5: P5091 does not affect p53 targets in MEC-1 cell line. A. MEC-1 cells were treated with P5091 at indicated concentrations for 24 hours, harvested and protein lysates were subjected to immunoblot analysis with anti-BAX, anti-puma, anti-NOXA, or anti-TUBULIN antibodies. **B.** Western blot analysis of PTEN levels after treatment with P5091 at indicated concentrations for 24 hours.

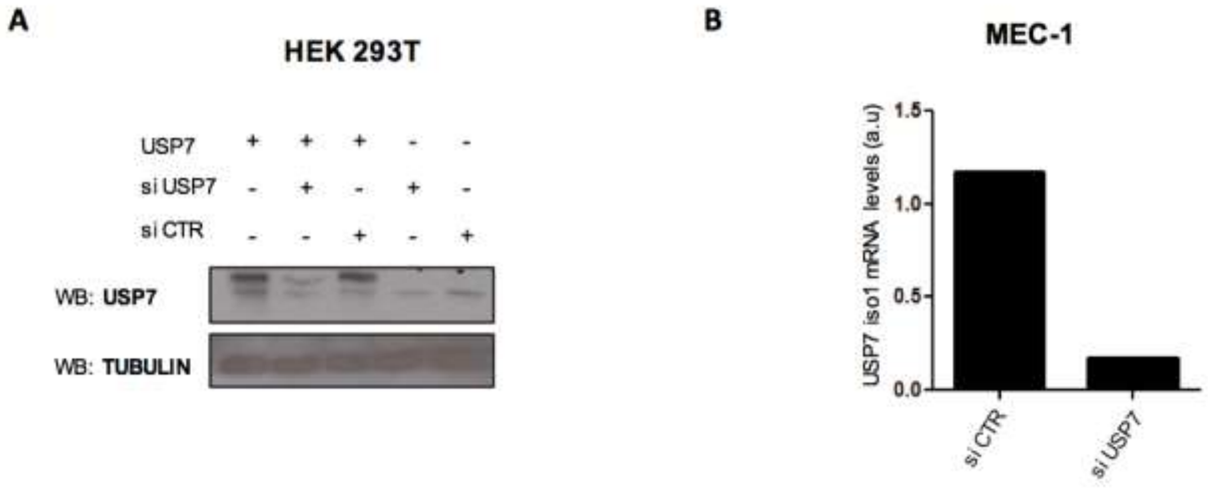

Supplementary Figure S6: siRNAs are able to efficiently silence USP7. A. Western blot analysis of the indicated proteins in HEK293T_upon transfection with USP7 siRNA. **B.** Real-time PCR analysis of mRNA USP7 levels in MEC-1 transfected with siCTRL or siUSP7.

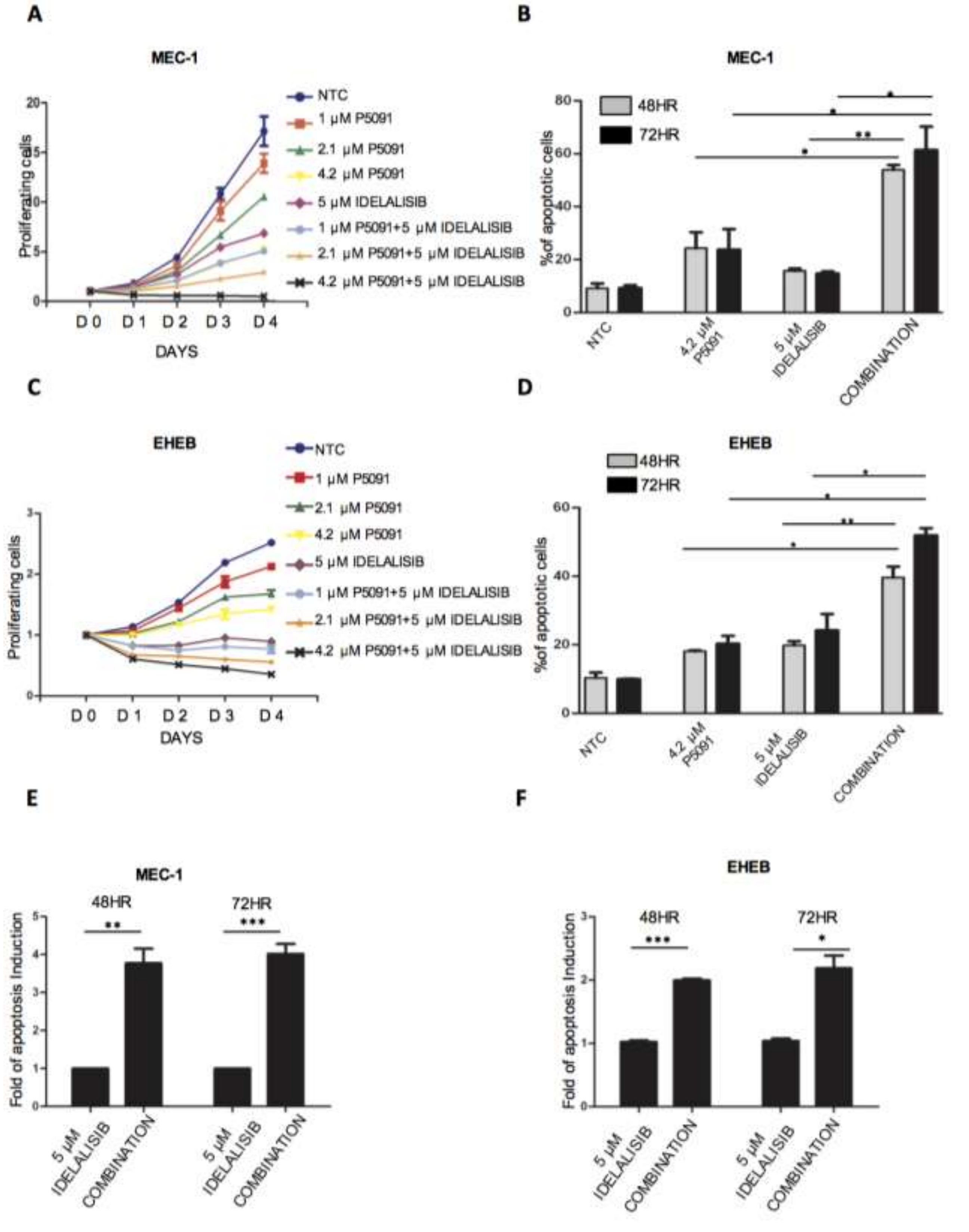

Supplementary Figure S7: Synergistic inhibitory effect of P5091 and Idelalisib treatment on CLL cell lines. A. Proliferation analysis of MEC-1 cells treated with USP7 inhibitor (P5091) and PI3K inhibitor (Idelalisib) for the indicated times and concentrations.

The number of cells at day 0 was set at 1 unit. **B.** Apoptosis analysis of MEC-1 cells treated with the indicated concentrations of P5091 and Idelalisib. *$p<0.05$; **$p<0.01$. **C.** Proliferation analysis of EHEB cells treated with USP7 inhibitor (P5091) and PI3K inhibitor (Idelalisib) for the indicated times and concentrations. The number of cells at day 0 was set at 1 unit. **D.** Apoptosis analysis of EHEB cells treated with the indicated concentrations of P5091 and Idelalisib. *$p<0.05$; **$p<0.01$. **E.** Mean of apoptosis fold-induction calculated on MEC-1 cells treated with P5091 and Idelalisib vs. Idelalisib alone. **$p<0.01$; ***$p<0.001$. **F.** Mean of apoptosis fold-induction calculated on EHEB cells treated with P5091 and Idelalisib vs Idelalisib alone

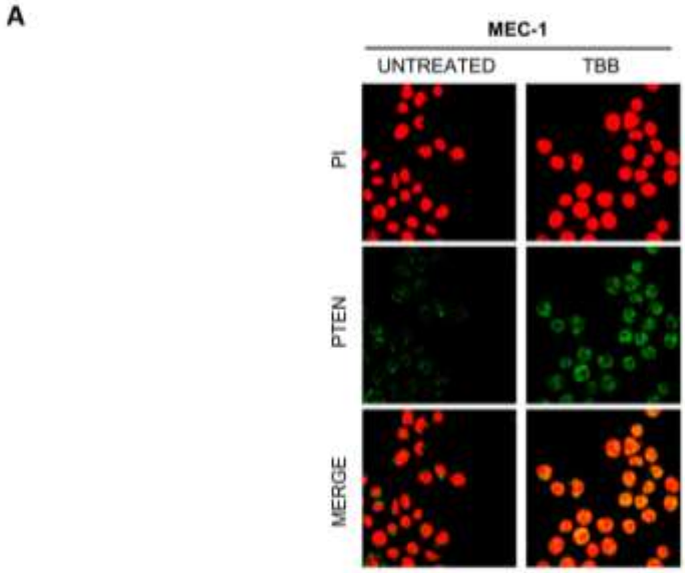

Supplementary Figure S8: CK2 inhibitors TBB promotes PTEN nuclear re-localization. A. Representative immunofluorescence images on MEC-1 cell line treated for 3hours with TBB and stained with anti-PTEN antibody (green). Propidium iodide (red) was used to visualize nuclei.

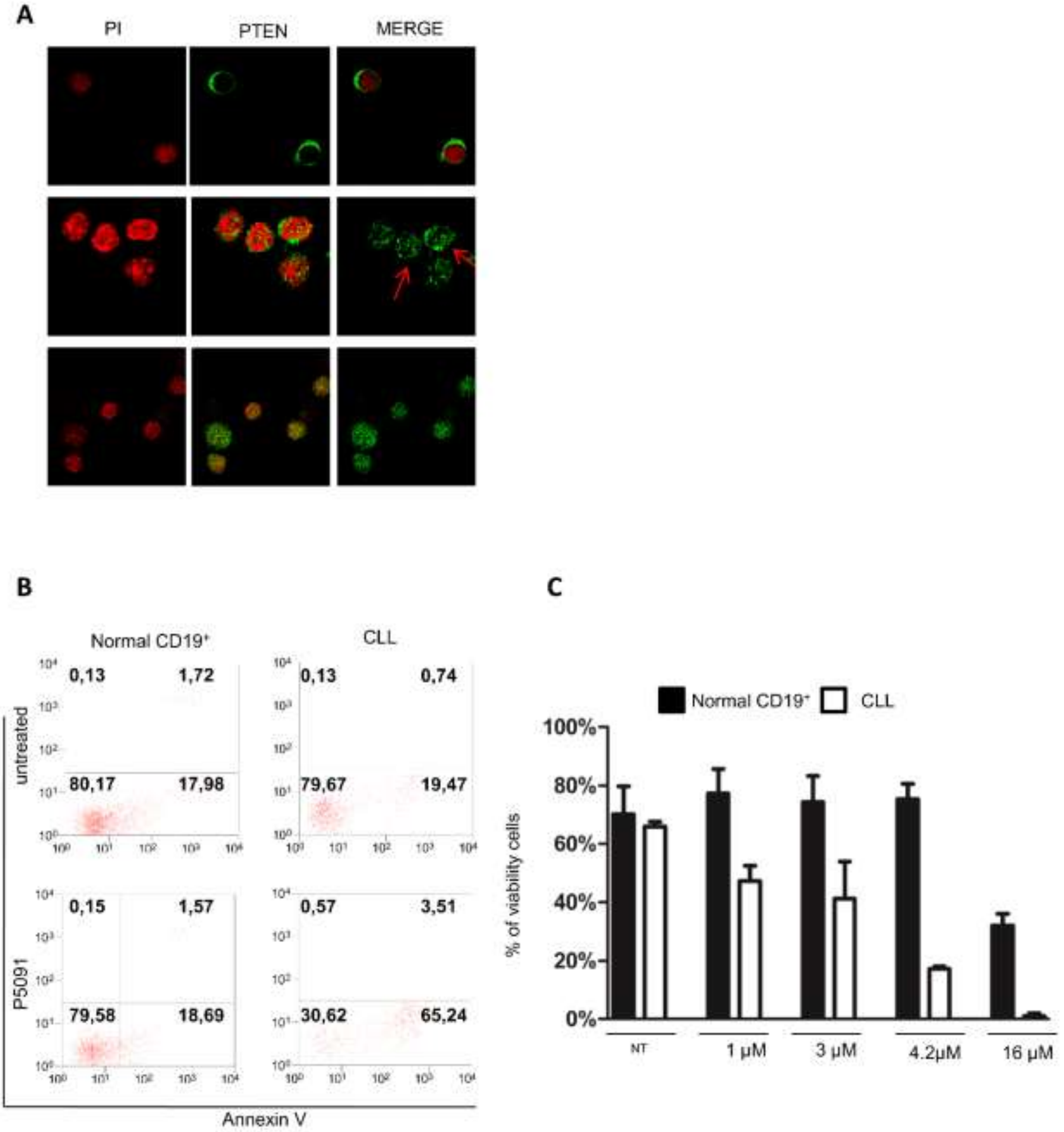

Supplementary Figure S9: PTEN cellular compartmentalization in primary CLL samples during P5091 treatment. A. Representative immunofluorescence images of PTEN compartmentalization in CLL primary cells. Upper panel represents a patient with an exclusively “cytosolic” pattern of PTEN compartmentalization. Middle panel represents a patient with a "predominantly cytosolic" pattern of PTEN compartmentalization. Lower panel represents a patient with a “diffuse” pattern of PTEN compartmentalization. **B.**

Representative Box-plot of CD19$^+$ lymphocytes collected from normal and CLL patients, treated with P5091 (4.2 µM) for 24 hours. Apoptosis was analyzed using Annexin V/PI staining assay **C.** CD19$^+$ lymphocytes from healthy donors were treated with indicated concentrations of P5091 for 24 hours, and cell viability was analyzed.
