# Supplementary material for: Therapeutic inhibition of USP7-PTEN network in chronic lymphocytic leukemia: a strategy to overcome TP53 mutated/deleted clones
Source: Oncotarget. 2017 Mar 17;8(22):35508–22. doi: 10.18632/oncotarget.16348 (PMC5482594; doi:10.18632/oncotarget.16348)
Supplement: Supplementary file 2 [file oncotarget-08-35508-s002.docx]

|  | **Anagraphic** | | | **Sample** | **CBC-PB (sampling)** | | | **Disease stage** | (prognosis) | | **Treatment** | | | | | **Cytogenetics (FISH)** | | | |
| --- | --- | --- | --- | --- | --- | --- | --- | --- | --- | --- | --- | --- | --- | --- | --- | --- | --- | --- | --- |
|  | Sex | DOB | Age (SMP) | [PB or BM] | WBC  ^1000/uL | NEU% | LYM% | RAI | CD38 | IgVH | **Treatment** | | | | | 12 | 11q23 | 17p13 | 13q14 |
| #1 | M | 28/03/1964 | 48 | PB | 75,10 | 4,0 | 89,0 | II | neg | mut | Y | RFC | 2014 | CR |  | wt | wt | wt |  |
| #2 | M | 23/06/1948 | 66 | PB | 159,00 | 3,8 | 93,2 | II | neg | n/a | Y | RFC | 2014 | CR |  | tri (23.1%) | wt | wt |  |
| #3 | F | 05/05/1932 | 82 | PB | 27,70 | 9,0 | 90,0 | IV | n/a | n/a | Y | RB | 2015 | CR |  | wt | wt | del (13.8%) |  |
| #4 | M | 12/08/1951 | 63 | PB | 143,00 | 4,9 | 91,7 | II | n/a | un  mut | Y | RFC | 2015 | PR | 2016 on PI3Ki | wt | wt | del (84.5%) |  |
| #5 | M | 10/11/1942 | 70 | PB | 71,10 | 7,1 | 90,8 | IV | n/a | n/a | Y | RFC | 2015 | CR |  | wt | wt | wt | del (88.2%) |
| #6 | M | 07/07/1946 | 66 | PB | 69,00 | n/a | 95,7 | 0 | neg | mut | N |  |  |  |  | wt | wt | wt |  |
| #7 | M | 27/01/1935 | 78 | PB | 63,40 | 6,2 | 92,3 | 0 | neg | mut | N |  |  |  |  | n/a | n/a | n/a |  |
| #8 | M | 23/12/1943 | 69 | PB | 76,60 | 6,7 | 91,1 | I | neg | mut | N |  |  |  |  | wt | wt | wt |  |
| #9 | M | 06/09/1961 | 51 | PB | 81,70 | 4,2 | 93,9 | IV | neg | mut | Y | RFC | 2014 | PR |  | wt | wt | wt |  |
| #10 | F | 26/07/1951 | 62 | PB | 11,80 | 27,0 | 65,0 | 0 | neg | mut | N |  |  |  |  | wt | wt | wt |  |
| #11 | M | 21/04/1964 | 49 | PB | 11,50 | 32,0 | 61,0 | 0 | neg | n/a | N |  |  |  |  | wt | wt | wt |  |
| #12 | F | 20/03/1935 | 81 | PB | n/a | n/a | n/a | n/a | n/a | n/a | n/a |  |  |  |  | wt | wt | wt |  |
| #13 | M | 09/11/1947 | 69 | PB | n/a | n/a | n/a | n/a | n/a | n/a | n/a |  |  |  |  | wt | wt | wt |  |
| #14 | M | 02/05/1957 | 59 | PB | n/a | n/a | n/a | n/a | n/a | n/a | n/a |  |  |  |  | wt | wt | wt |  |
| #15 | M | 08/12/1943 | 71 | PB | 57,40 | n/a | 92,0 | I | neg | n/a | N |  |  |  |  | n/a | n/a | n/a |  |
| #16 | M | 15/10/1938 | 75 | PB | 18,50 | n/a | 73,0 | II | pos | un  mut | Y | RFC | 2007 | CR |  | n/a | n/a | n/a |  |
| #17 | M | 15/01/1947 | 67 | PB | 8,29 | 45,0 | 46,0 | n/a | pos | mut | Y | FC-Campath | 2006 | CR |  | n/a | n/a | n/a |  |
| #18 | F | 27/10/1946 | 66 | PB | 45,60 | 9,2 | 87,8 | n/a | n/a | n/a | n/a |  |  |  |  | wt | wt | wt |  |
| #19 | F | 29/04/1950 | 66 | PB | n/a | n/a | n/a | n/a | n/a | n/a | N |  |  |  |  | wt | wt | wt |  |
| #20 | F | 25/07/1952 | 64 | PB | n/a | n/a | n/a | n/a | n/a | n/a | N |  |  |  |  | wt | wt | del |  |
